# In vitro antibiotic susceptibility and biofilm production of *Staphylococcus aureus* isolates recovered from bovine intramammary infections that persisted or not following extended therapies with cephapirin, pirlimycin or ceftiofur

**DOI:** 10.1186/s13567-017-0463-0

**Published:** 2017-09-21

**Authors:** Céline Ster, Valérie Lebeau, Julia Leclerc, Alexandre Fugère, Koui A. Veh, Jean-Philippe Roy, François Malouin

**Affiliations:** 1 Centre d’Étude et de Valorisation de la Diversité Microbienne (CEVDM), Département de Biologie, Faculté des Sciences, Université de Sherbrooke, Sherbrooke, QC J1K 2R1 Canada; 2 Département de Sciences Cliniques, Faculté de Médecine Vétérinaire, Université de Montréal, C.P. 5000, St-Hyacinthe, QC J2S 7C6 Canada

## Abstract

*Staphylococcus aureus* intramammary infections (IMIs) have low cure rates using standard antibiotic treatment and increasing the duration of treatment usually improves therapeutic success. Chronic IMIs are thought to be caused by bacteria presenting a specific virulence phenotype that includes the capacity to produce greater amounts of biofilm. In this study, antibiotic susceptibility and biofilm production by *S. aureus* isolates recovered from IMIs that were cured or not following an extended therapy with cephapirin, pirlimycin or ceftiofur for 5, 8 and 8 days, respectively, were compared. An isolate was confirmed as from a persistent case (not cured) if the same *S. aureus* strain was isolated before and after treatment as revealed by the same VNTR profile (variable number of tandem repeats detected by multiplex PCR). The antibiotic minimal inhibitory concentrations (MICs) were determined for these isolates as well as the capacity of the isolates to produce biofilm. Isolates from persistent cases after extended therapy with cephapirin or ceftiofur had higher MICs for these drugs compared to isolates from non-persistent cases (*p* < 0.05) even though the antibiotic susceptibility breakpoints were not exceeded. Isolates of the ceftiofur study significantly increased their biofilm production in presence of a sub-MIC of ceftiofur (*p* < 0.05), whereas isolates from the pirlimycin group produced significantly less biofilm in presence of a sub-MIC of pirlimycin (*p* < 0.001). Relative antibiotic susceptibility of the isolates as well as biofilm production may play a role in the failure of extended therapies. On the other hand, some antibiotics may counteract biofilm formation and improve cure rates.

**Electronic supplementary material:**

The online version of this article (doi:10.1186/s13567-017-0463-0) contains supplementary material, which is available to authorized users.

## Introduction

*Staphylococcus aureus* (*S. aureus*) is a major bacterial pathogen causing intramammary infections (IMIs) [1] and is most often responsible for a chronic and contagious mastitis that is difficult to treat with antibiotics [2]. Reported cure rates for *S. aureus* mastitis are usually low but vary from 4 to 92% and seem to depend on many host and bacterial factors such as cow parity, level of somatic cell counts or the genetic background of *S. aureus* isolates and their ability to produce biofilm [2, 3]. Biofilm is an extracellular matrix in which bacteria are more resistant to antibiotics, the immune system or disinfectants [4]. It is commonly believed that the discrepancy between the antimicrobial susceptibility in vitro and cure rate is related to the capacity of *S. aureus* to produce biofilm during IMIs [5].

Cephapirin, pirlimycin and ceftiofur are antibiotics available for the treatment of bovine mastitis in North America [6]. Cephapirin is a first generation cephalosporin antibiotic while ceftiofur is a third generation. Cephalosporins (part of the β-lactam class) inhibit bacterial transpeptidases, which are responsible for cell wall peptidoglycan biosynthesis [7]. Pirlimycin belongs to the lincosamide class of antibacterial agents [8]. It acts by inhibiting bacterial protein synthesis via binding to the 50S subunit of the ribosome.

Extending the duration of antibiotic treatment seems to improve therapeutic success [2, 6]. In a study evaluating the efficacy of a 5-day cephapirin treatment of chronic subclinical *S. aureus* IMIs, cow bacteriological cure rates were of 25.8 and 3.3% for the treated and untreated control groups, respectively [9]. In another study comparing 2-, 5- and 8-day therapies with pirlimycin, quarter bacteriological cure rates were of 13.3, 31.3 and 83.3%, respectively, while no cure (0%) for untreated controls was observed [10]. Several ceftiofur treatment durations for subclinical *S. aureus* IMIs were compared in a study by Oliver et al. [7]. Bacteriological cure rates were of 36, 17, 7 and 0% for the 8-, 5-, 2-day ceftiofur treatment and for the untreated control groups, respectively. A more recent study also assessed the impact of an extended therapy using ceftiofur for the treatment of clinical mastitis caused by *S. aureus* IMIs. A quarter bacteriological cure rate of 0% was observed for the group of cows treated for 2 days (conventional treatment) while a bacteriological cure rate of 47.4% was observed for cows treated for 8 days [11].

It is thus apparent that extended therapies increase cure rates for IMIs caused by *S. aureus*. However, not all *S. aureus* IMIs are cured and cure rates vary based on the type of antibiotic treatment used. This study aimed at making associations between *S. aureus* biofilm formation, antibiotic susceptibility and the success or failure of extended therapies with cephapirin, pirlimycin or ceftiofur.

## Materials and methods

### Extended antibiotic therapy

For the purpose of this study, we used *S. aureus* strains that were collected in three different field studies in which extended therapies were used for treatment of *S. aureus* IMIs. *S. aureus* isolates were identified from milk samples as Gram-positive cocci showing a positive catalase test, hemolysis on blood agar, and positive nuclease and coagulase tests.

An IMI was confirmed if at least one colony of *S. aureus* from a 10-µL milk sample (i.e., ≥ 100 cfu/mL) was found. Only one isolate was kept from each sample. Since only 10 µL milk samples were initially plated to assess the bacterial content, it was assumed that this isolate was predominant but IMI caused by multiple isolates each present in large numbers cannot be ruled out. Each of the three field studies are briefly described below.

#### Extended therapy with cephapirin

The *S. aureus* isolates were collected during a study that assessed the efficacy of an extended therapy with cephapirin against chronic subclinical IMIs [9]. Briefly, 14 herds from a group of dairy herds in the Saint-Hyacinthe region of the Province of Quebec (Canada) were enrolled in this study. Milk samples were collected from dairy cows with a history of chronic IMIs caused by *S. aureus*. When *S. aureus* IMI was confirmed based on the presence of the bacterium in milk, cephapirin (200 mg/dose, Cefa-Lak®, Boehringer Ingelheim, Burlington, ON, Canada) was administered in all 4 quarters at each milking for 5 consecutive days and 3 milk samples were taken 10, 24 and 31 days after treatment for bacteriological analysis. The labelled treatment for cephapirin is 2 doses 12 h apart (2 consecutive milkings). Among the 29 cases analyzed in this study, 12 cases (41.4%) showed bacteriological cure, i.e., showed 3 consecutive negative milk samples after the end of the extended therapy (Table 1). For the other 17 cases, *S. aureus* was isolated from at least one of the 3 samplings collected after the end of the treatment, indicating a failure of the extended therapy (i.e., persistent cases based on bacteriology, Table 1).

**Table 1:** **Overview** ***S. aureus*** **isolates collected in three different field studies in which extended therapies were used for treatment of IMIs**

	Extended therapy with		
	Cephapirin	Pirlimycin	Ceftiofur
Total number of cases^a^	29	40	17
Based on bacteriology			
Cases in which *S. aureus* was eliminated	12	33	9
Cases in which *S. aureus* was isolated after therapy	17	7	8
Proportion of isolates that were eliminated (%)	41.3	82.5	52.9
Based on VNTR analysis			
Isolates that were eliminated or that were distinct by VNTR before and after therapy	19	34	9
Isolates from persistent cases (same VNTR before and after therapy)	10	6	8
Proportion of isolates that were eliminated (%)	65.5	85.0	52.9
Number of isolates characterized in this study^b^			
Isolates from cured cases	12	23^c^	9
Isolates from persistent cases (VNTR-confirmed)^c^	10	6	8

^a^Cases are quarters infected with *S. aureus*. Isolates from cured cases are revealed when one *S. aureus* isolate is found before treatment but no *S. aureus* is detected after the end of treatment. Persistent cases are revealed when one *S. aureus* isolate is found before treatment and at least one *S. aureus* isolate is also found after the end of treatment.

^b^Cases that were not validated as persistent (possible new infections by a different *S. aureus* isolate) were excluded of the study. When multiple isolates were collected from the same cow (different quarters), only one isolate was selected for the study.

^c^Only the isolates collected before treatment were used for the rest of the study.

#### Extended therapy with pirlimycin

The bacterial isolates were collected from primiparous cows with *S. aureus* IMIs that were treated in the first week of lactation with pirlimycin (50 mg/dose, once a day, Pirsue^®^, Zoetis, Kirkland, QC, Canada) in the affected quarters for 8 consecutive days. The labelled treatment for pirlimycin in Canada is 2 or 8 doses 24 h apart. The cows were coming from 23 herds in the Drummondville region of the Province of Québec (Canada) and this treatment protocol was part of the usual udder health control program on these herds. Treatment success or failure was assessed by bacteriological milk cultures of quarter milk samples collected 3–8 weeks after treatment (average of 32 days). Forty quarters from 36 cows were subjected to this treatment regimen and 82.5% of *S. aureus* isolates (i.e., from 33 quarters) were from successful therapy based on bacteriology (Table 1). In 7 cases, *S. aureus* was isolated after treatment indicating therapeutic failure (i.e., 7 persistent cases based on bacteriology, Table 1). Due to the large number of isolates from cured cases, we only randomly included a total of 23 distinct isolates from cured cases (on a possibility of 33) for further characterization in this study (as indicated in Table 1).

#### Extended therapy with ceftiofur

*Staphylococcus aureus* isolates were collected during a study that assessed the efficacy of an extended therapy with ceftiofur for the treatment of mild to moderate clinical mastitis [11]. Labelled treatment in Canada is 2 doses 24 h apart. Briefly, 22 herds located in the Province of Quebec and Eastern Ontario (Canada) were enrolled in this study. A total of 17 *S. aureus* isolates from 17 cases treated with an extended therapy with ceftiofur for 8 consecutive days (125 mg/dose, once a day, Spectramast^®^ LC, Pfizer Animal Health, Kirkland, QC) were available for analysis. Milk samples were taken 7, 14 and 21 days after the end of the treatment to assess bacteriological cure. Based on bacteriological results, 8 cases responded to treatment (52.9%, Table 1), whereas in 9 cases, *S. aureus* was isolated in at least one of the milk samples collected after the end of treatment, indicating therapeutic failure.

### VNTR analysis

VNTR analysis (variable number of tandem repeats) was used to validate bacterial persistence of the same *S. aureus* strain before and after the extended therapy. First, genomic DNA of the different isolates was purified using the Gene Elute kit according to the recommendations of the manufacturer (Sigma Aldrich, Oakville, ON, Canada). Then, VNTR analysis for five genes (*sdr*, *clfA*, *clfB*, *ssp* and *spa*) was performed by multiplex PCR as previously described by Sabat et al. [12]. Isolates from each case were processed at the same time (same PCR mix) and their PCR products were migrated on the same electrophoresis agarose gel. Pattern of the isolates, recovered before and after treatment, from a same case were compared visually to assess their similarity. As described by Veh et al. [13], a control strain (SHY97-3906) was added to each multiplex PCR batch and electrophoresis gel to ensure the repeatability of the method.

This VNTR analysis aimed at excluding the possible cases of a cure followed by a new infection with a distinct *S. aureus* isolate or also cases in which multiple strains could be involved. Isolates before and after extended therapy were considered as the same strain, from a persistent case, if their electrophoretic VNTR profiles were identical. If the isolates before and after extended therapy showed different VNTR profiles, this case was excluded from the study.

### Antibiotic minimal inhibitory concentration

The minimal inhibitory concentrations (MICs) of cephapirin, pirlimycin and ceftiofur for all isolates were determined by a 96-well plate broth microdilution technique, following the recommendations of the Clinical Laboratory Standards Institute (CLSI) [14]. Briefly, the antibiotics were serially diluted (doubling dilutions) in Mueller–Hinton broth (cation adjusted, CAMHB; BD, Mississauga, ON) within a 96-well plate before the same volume of the bacterial inoculum was added to each well. The inoculum was prepared from an overnight culture in CAMHB, first diluted to a 0.5-McFarland standard (≈1.5 × 10^8^ CFU/mL) before adding ≈10^5^–10^6^ CFU/mL to each well. The *S. aureus* strain ATCC29213 was used as a quality control as recommended by Clinical Laboratory Standards Institute (CLSI). The MIC was the lowest concentration of antibiotic preventing growth. Susceptibility thresholds used were 8, 2 and 2 μg/mL for cephapirin, pirlimycin and ceftiofur, respectively. Resistance breakpoints used were 32, 4 and 8 µg/mL for cephapirin, pirlimycin and ceftiofur, respectively.

### Biofilm production

Biofilm formation was evaluated by spectrophotometry using crystal violet staining, as previously described with few modifications [13, 15]. Briefly, isolates were cultured from frozen stocks onto tryptic soy agar plates and incubated at 35 °C overnight. Colonies were then inoculated into brain heart infusion containing 0.25% of glucose (Sigma Aldrich) to obtain a 0.5-McFarland standard (≈1.5 × 10^8^ CFU/mL). The suspension was then transferred into wells of a flat bottom polystyrene microtiter plates containing half the volume of the same medium. The plates were incubated at 35 °C for 24 h, without agitation and under aerobic conditions. The supernatant was then discarded and the wells were delicately washed three times with 200 μL of PBS. The plates were dried, and then stained for 30 min with crystal violet (Sigma Aldrich). Wells were then washed twice with 200 μL of water and allowed to dry again. A volume of 200 μL of 95% ethanol was added to each well and plates were incubated at room temperature for 1 h with frequent agitation. The absorbance of each well was then measured at 560 nm using a plate reader (Epoch, Bio-Tek instruments, Vinooski, VT). The *S. aureus* reference strain Newbould (ATCC 29740), initially isolated from a bovine mastitis case, was included into each plate to normalize for plate-to-plate variations. This strain is a moderate biofilm producer with an average OD 560 nn value of 0.15 in biofilm assays. Biofilm measurements were averages of three independent experiments performed on different days. Each independent experiment included four wells for each strain (four technical replicates). Average biofilm production for each isolate in each plate was calculated and normalized by dividing the average biofilm production of the isolates by the average production of the reference train. Then, the normalized measured biofilm enumeration of each isolate obtained from the three independent assays were combined.

To evaluate the effect of a subinhibitory concentration of cephapirin, pirlimycin or ceftiofur on biofilm production, measurements were also performed for each isolate grown in the presence of a sub-MIC (0.25 × MIC) of those antibiotics for the isolate. For example, if a strain had a MIC of 1 µg/mL for pirlimycin, biofilm assay was performed with 0.25 µg/mL of pirlimycin. The concentration was thus adjusted for each isolate and for each antibiotic.

### Statistical analysis

Statistical analyses were performed using the GraphPad Prism software (v5.00). The distribution of the isolates from cured cases vs the isolates from persistent cases according to their MIC for an antibiotic was compared using a Chi square trend test. Biofilm production for the different groups of isolates was compared with the Kruskal–Wallis test (nonparametric one way analysis of variance) followed by a Dunn’s multiple comparisons test. The biofilm production of the isolates from cured cases vs the isolates from persistent cases and biofilm production in presence or absence of a sub-MIC of antibiotics were compared with the Mann–Whitney test (nonparametric *t* test). Statistical tests used for the analysis of each of the experiments are specified in the Figure legends. Differences were considered statistically significant when *p* < 0.05.

## Results

### VNTR analysis of the isolates and validation of persistent IMI cases

VNTR analysis was performed to validate IMI persistence despite extended therapy. A persistent case was validated when the *S. aureus* isolates collected before and after the extended therapy showed an identical VNTR electrophoretic profile (see Additional file 1 for examples). Among the IMIs that were classified as persistent based on the bacteriological analyses of the milk samples, 10/17, 6/7 and 8/8 of the IMIs from the extended therapy with cephapirin, pirlimycin and ceftiofur, respectively, were confirmed as persistent after VNTR analysis (see the number of isolates from persistent cases based on VNTR analysis, Table 1). For each pair of isolates from persistent cases (before and after therapy), only the results obtained for the “before therapy isolate” are presented here as no difference in VNTR, MICs or biofilm production among the pair members was observed (data not shown). Isolates from the non-validated cases of persistence (i.e., showing different VNTR profiles before and after therapy) were excluded for the rest of this study as these non-validated cases might represent cases where the quarter was successfully treated by the extended therapy but then re-infected by a new isolate having a different VNTR profile. The non-validated cases might also have represented cases in which multiple strains were involved. The data available about those cases did not permit to select the most likely hypothesis and these non-validated cases were thus excluded from the study.

### Antibiotic MICs among the study groups

**Figure 1:**
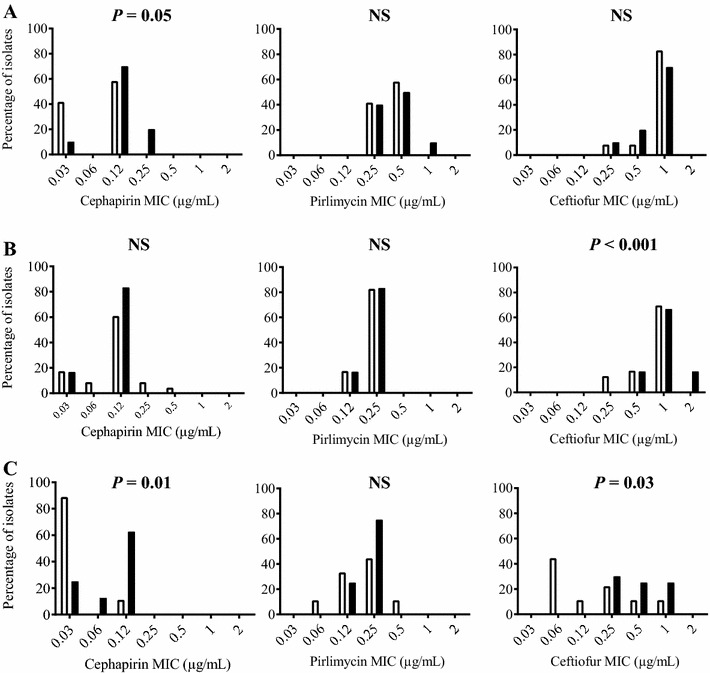
**Distribution of antibiotic MICs for isolates that were from cured or persistent cases.** Isolates from cured cases were represented with open bars while isolates from persistent cases are represented with closed bars. Isolates were collected from an extended therapy study with cephapirin (**A**), pirlimycin (**B**) or ceftiofur (**C**). The distribution of the isolates from cured vs the persistent cases according to their MIC for an antibiotic was compared using a Chi square trend test: NS, not statistically significant.

Antibiotic resistance of bacterial isolates may cause failure of antibiotic therapy (standard or extended). Here, no difference in the antibiotic MIC_50_ or MIC_90_ (MIC that inhibits the growth of 50 or 90% of the tested strains, respectively) was observed between the isolates from cured and persistent cases for the three extended therapies. Cephapirin MIC_50_ and MIC_90_ values were of 0.12 and 0.25 µg/mL, respectively, for isolates of the cephapirin study, pirlimycin MIC_50_ and MIC_90_ were 0.25 and 0.25 µg/mL for isolates of the pirlimycin study, and ceftiofur MIC_50_ and MIC_90_ were 0.25 and 1 µg/mL for isolates of the ceftiofur study. Based on CLSI susceptibility thresholds of 8, 2 and 2 μg/mL for cephapirin, pirlimycin and ceftiofur, respectively (resistance breakpoints are 32, 4 and 8 µg/mL respectively), none of the isolates could be considered as resistant in vitro. However, the distribution of the antibiotic MICs for the isolates from cured and persistent cases was different within the three study groups (i.e., extended therapies to cephapirin, pirlimycin and ceftiofur) (Figure 1). This comparison demonstrates that the *S. aureus* isolate populations collected from those three studies were different. Noteworthy, cephapirin and ceftiofur generally had higher MICs for the isolates from the persistent cases in two of the three extended therapy studies, whereas the distribution of pirlimycin MICs for the isolates from cured and persistent cases did not differ in any of the three studies (Figure 1). Note that the isolates from the persistent cases tested were the “before therapy isolates” as mentioned in “Materials and methods” section and some of the observed differences in antibiotic susceptibility could therefore not have resulted from a selective pressure by the therapy under investigation.

### Biofilm production

**Figure 2:**
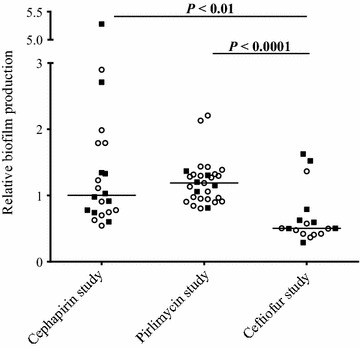
**Biofilm production for isolates collected from the different extended therapy studies.** Biofilm production of the different isolates was normalized with the biofilm production of *S. aureus* strain Newbould. Isolates from cured cases are represented with open circles while isolates from persistent cases are represented with closed squares. Horizontal bars represent the medians. Statistical analysis: Kruskal–Wallis test with Dunn’s multiple comparisons test.

The *S. aureus* isolates collected from the three different therapy studies were also distinct based on their in vitro biofilm production. The isolates from the ceftiofur study produced significantly less biofilm than isolates from the two other studies although for each of the study groups, there was no difference in biofilm production between isolates from cured or persistent cases (Figure 2).

**Figure 3:**
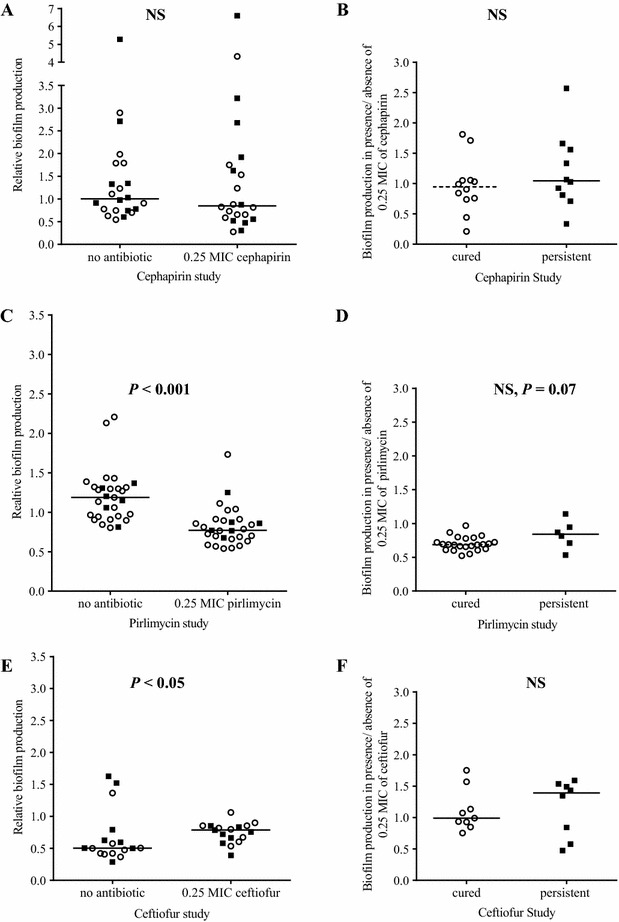
**Biofilm production in presence or absence of the sub-MIC of the different antibiotics. A**, **B** *S. aureus* isolates collected from the cephapirin study. **C**, **D** *S. aureus* isolates collected from the pirlimycin study; and **E**, **F** *S. aureus* isolates collected from the ceftiofur study. For all graphs, open circles represent data for isolates from cured cases and closed squares represent data for isolates from persistent cases. Horizontal bars represent the medians. **A**, **C**, and **E** present biofilm production (relative to the *S. aureus* strain Newbould) for each of the isolates grown in presence or absence of the sub-MIC (0.25 × MIC) of cephapirin, pirlimycin or ceftiofur for that isolate, respectively. **B**, **D**, **F** present the biofilm production of each of the isolates from cured or persistent cases as determined in the presence of the sub-MIC of cephapirin, pirlimycin or ceftiofur, respectively, relative to the biofilm production of the same isolate in absence of antibiotic. Statistical analysis: Mann–Whitney: NS, not statistically significant.

Biofilm production by each isolate was also evaluated in presence of a sub-MIC (0.25 × MIC) of the different antibiotics previously determined against each of the isolates (Figure 3). No difference in biofilm production was observed in presence or absence of the sub-MIC of cephapirin for the entire set of isolates collected in the cephapirin study (Figure 3A). Also, taken individually, each of the cephapirin-therapy isolates from cured or persistent cases showed the same ability to produce biofilm in presence or absence of the sub-MIC of cephapirin (Figure 3B). On the other hand, isolates from the extended therapy with pirlimycin produced significantly less biofilm in presence of a sub-MIC of pirlimycin (*p* < 0.0001, Figure 3C). Even though the group of isolates from the extended therapy with ceftiofur produced significantly less biofilm than the two other studied groups (Figure 2), the presence of a sub-MIC of ceftiofur significantly increased the biofilm production of these isolates (*p* < 0.05, Figure 3E). Finally, there was a tendency for the isolates from persistent cases from the pirlimycin study to produce more biofilm than isolates from the cured cases in presence of the sub-MIC of the antibiotic (*p* = 0.07, Figure 3D), but like for the isolates from the persistent cases of the ceftiofur study (Figure 3F), this difference was not statistically significant.

## Discussion

The incidence of new IMI may increase with duration of intramammary treatments [2]. Indeed, some studies that have evaluated the efficacy of extended antibiotherapies reported an increase in new IMI rates [10, 11, 16]. It was reported that new infections with *E. coli* or *Klebsiella* can occur during extended therapy. New IMIs can also occur between the end of a treatment and post-treatment samplings, which may appear as a therapeutic failure. In the present study, we used VNTR analysis to validate persistent cases. We found that some cases that were initially considered persistent IMIs based on the bacteriological analysis of milk samples needed to be re-classified as either potentially cured and followed by a new infection or cases in which multiple strains were involved since post-therapy samplings revealed *S. aureus* isolates having different VNTR profiles. VNTR analysis is therefore a useful tool to quickly discriminate between a persistent case and more complex cases where distinct strains are observed. VNTR analysis was also used to discriminate epidemic strains from sporadic strains in hospitals [17]. The effectiveness of VNTR as a molecular typing tool make VNTR analysis highly comparable to more complex methods such as PFGE and MLST [12, 18]. VNTR analysis can thus provide information on the diversity of isolates present in a herd, identify predominant clones and help management of IMIs [2].

Several differences other than the antibiotic treatment prevent a direct comparison of these therapies. First, case definitions and definition of cure were different. In the study using cephapirin [9], the *S. aureus* IMI cases were chronic subclinical infections and cure was defined using three milk cultures at 10, 24 and 31 days post-treatment. In the study using ceftiofur [11], *S. aureus* isolates were collected from clinical mastitis cases and cure was defined using three milk cultures at 7, 14 and 21 days post-treatment. Finally, the study with pirlimycin (unpublished) used first or second lactation cows at calving and only one milk culture between 3 and 8 weeks post-treatment was used for cure definition. Also, the three antibiotic treatments were not applied on the same population of cows and herd level factors may certainly affect cure rates. Finally, the bacterial isolate populations collected from the cephapirin, ceftiofur and pirlimycin studies also differed. The distribution of antibiotic MICs (Figure 1) and the overall differences in biofilm production for the three isolate populations (Figure 2) attest that bacterial factors may affect the outcome of the therapy.

The specific aim of the present study was indeed to examine some of the bacterial factors such as biofilm production that could influence or explain the relative success of extended therapies with cephapirin, pirlimycin or ceftiofur. By comparing biofilm formation and antibiotic susceptibility of the *S. aureus* isolates from cured and persistent cases we could suggest some explanations for failure or success of therapy as discussed below.

The cephapirin and ceftiofur MICs for the isolates from cases that persisted to the extended therapy with these antibiotics were generally higher than the MICs determined for the isolates from the cured cases. Although the isolates from persistent cases can still be considered as clinically susceptible to cephapirin (MIC ≤ 8 μg/mL) or ceftiofur (MIC ≤ 2 μg/mL), the higher MICs generally found for these isolates compared to the isolates from the cured cases (Figures 1A and 1C, respectively) may improve their capacity to persist in the mammary gland. The mammary gland is a large organ and intramammary antibiotic diffusion might not be homogenous [2]. A slight decrease in antibiotic activity (i.e., slightly higher MICs) against specific *S. aureus* strains and an unequal tissue distribution of the antibiotic may result in a persistent case. Then again, another study with cephapirin could not demonstrate a correlation between in vitro susceptibility test results (MICs) and the outcome of therapy [19], although this was a study on multiple Gram-positive pathogens and the standard prescribed cephapirin therapy was used.

Biofilms offer protection from antibiotic activity and host defenses and is one of the causes often proposed for therapeutic failure [2, 4, 20]. Interestingly, we report here that *S. aureus* isolates from both cured and persistent cases are producing significantly more biofilm in the presence of a sub-MIC of ceftiofur (Figure 3E). Noteworthy however, there were at least 3 of the 17 strains that showed less biofilm production in presence of this antibiotic and this indicated that there were strain to strain variations in the response to this antibiotic (Figure 3E). This sub-MIC effect on biofilm production, combined to the higher MICs of the ceftiofur-persistent isolates mentioned above may both have contributed to treatment failure. The mechanism responsible for the increase biofilm production in the presence of a sub-MIC of ceftiofur is not known at this time but it is possible that the damage produced to the bacterial cell wall by this β-lactam antibiotic activates a sigma factor B-dependent stress response that helps biofilm formation [21]. On the other hand, as far as pirlimycin is concerned, we observed no difference in the degree of susceptibility of the *S. aureus* isolates collected from cases that persisted or that were cured by the extended therapy (Figure 1B), and remarkably, the presence of a sub-MIC of pirlimycin significantly reduced biofilm production (Figure 3C). The cure rates of pirlimycin may have been helped by such conditions. Extended pirlimycin treatments with cure rates up to 86% were also previously reported [7, 9, 10, 22, 23]. Pirlimycin is a lincosamide antibiotic that blocks proteins synthesis. Huang et al. [24] showed that clindamycin, another lincosamide, can also reduce the biofilm surface of *S. aureus*. Rachid et al. [25] showed that subinhibitory concentrations of clindamycin had no effect on the expression of the *ica* operon (involved in biofilm production) in *S. epidermidis* during biofilm formation. However, using *S. epidermidis* cells embedded in a biofilm, Gomes et al. [26], showed that the clindamycin–rifampicin combination reduces the expression of genes *icaA* and *rsbU* (two genes involved in biofilm formation) compared to that is observed with rifampicin alone.

Here, we have measured the impact of sub-inhibitory concentrations of antibiotics on the capacity of *S. aureus* to produce biofilm. Another interesting approach that may have facilitated the evaluation of the effect of biofilm production on antibiotic action and associations with treatment efficacy was proposed by Melchior et al. [27], i.e., the use of an antibiotic susceptibility assay for bacteria embedded in biofilms and the determination of the minimum concentration of biofilm eradication (MBEC) [5]. This would have provided information on the impact of antibiotics on an already formed biofilm. These in vitro methods address two different aspects of the possible action of antibiotics on biofilms and vice versa. It is still however very difficult to predict the success of treatment using in vitro tests.

Each antibiotic possesses different physical and biological properties (solubility, protein binding, absorption, tissue distribution, half-life, MIC, etc.) that certainly influence therapeutic efficacy. The host (cow) can also influence the outcome of treatment. For examples, cow parity and the position of the infected quarter (rear vs front) can affect cure rates [2]. Therefore, this study could not take in account all these factors but for each study group (i.e., each extended therapy taken individually), the study and comparison of both isolates from cured and persistent cases revealed some interesting bacterial factors and antibiotic effects that may influence the therapeutic outcome. The generic representation we made of the observed biological responses may however not be valid for a proportion of *S. aureus* isolates due to strain-to-strain variations. More work is needed to determine all host and bacterial factors, in addition to the pharmacological factors, involved in the success and failure of extended therapies for bovine *S. aureus* IMIs.

## Additional file

**Additional file 1.** **Examples of VNTR profiles for bacteriology-defined persistent cases from the cephapirin therapy study.** S1 represents the VNTR profile of the *S. aureus* isolate before treatment with cephapirin while S2 represents the VNTR profile of the *S. aureus* isolate recovered after treatment. The asterisk (*) shows identical VNTR profile, i.e., the VNTR-validated persistent cases that were investigated in this study. The arrows indicate some of the differences between two profiles, and as such, the isolate recovered after treatment was considered to be the result of a new infection; the case was considered neither cured or persistent and was therefore not evaluated in this work.
