# Supplementary figures and images for: In vitro antibiotic susceptibility and biofilm production of Staphylococcus aureus isolates recovered from bovine intramammary infections that persisted or not following extended therapies with cephapirin, pirlimycin or ceftiofur
Source: Vet Res. 2017 Sep 21;48:56. doi: 10.1186/s13567-017-0463-0 (PMC5609010; doi:10.1186/s13567-017-0463-0)

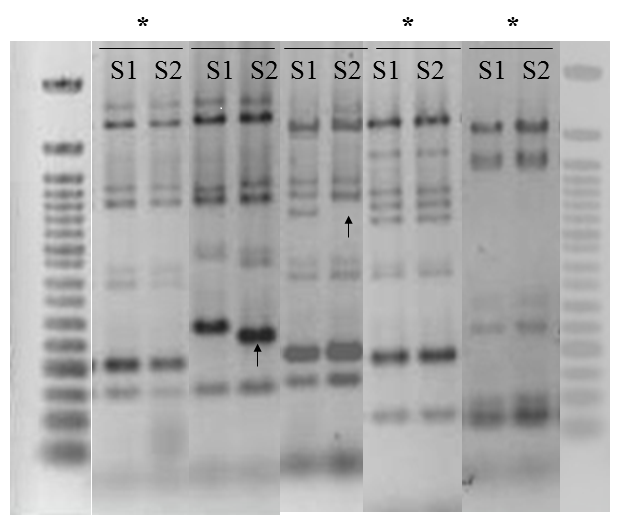

Supplement: Supplementary file 1 — Additional file 1. Examples of VNTR profiles for bacteriology-defined persistent cases from the cephapirin therapy study. S1 represents the VNTR profile of the S. aureus isolate before treatment with cephapirin while S2 represents the VNTR profile of the S. aureus isolate recovered after treatment. The asterisk (*) shows identical VNTR profile, i.e., the VNTR-validated persistent cases that were investigated in this study. The arrows indicate some of the differences between two profiles, and as such, the isolate recovered after treatment was considered to be the result of a new infection; the case was considered neither cured or persistent and was therefore not evaluated in this work. [file 13567_2017_463_MOESM1_ESM.tif]
